# Supplementary material for: AI-augmented differential diagnosis of granulomatous rosacea and lupus miliaris disseminatus faciei: A 23–year retrospective pilot study
Source: PLoS One. 2025 Jun 30;20(6):e0326763. doi: 10.1371/journal.pone.0326763 (PMC12208491; doi:10.1371/journal.pone.0326763)
Supplement: S1 Table — (DOCX) [file pone.0326763.s001.docx]

**Supporting Information**

**S1 Table. Details of Neural Network Models used in this Study**

| **^†^Model** | **Depth** | **Input size** |
| --- | --- | --- |
| **ResNet50** | 50 layers (49 Convolutional + 1 Fully Connected layer) with 4 residual blocks | 224×224 |
| **Inception-V3** | 48 Convolutional + 1 Global Average Pooling Layer using factorised convolutions | 299×299 |
| **Xception** | 71 Convolutional + 1 Global Average Pooling Layer with Inception module using depthwise separable convolutions | 299×299 |
| **DenseNet169** | 169 Convolutional + 1 Global Average Pooling + 1 Fully Connected layer with densely connected block | 224×224 |
| **EfficientNet-B0** | 18 Convolutional + 1 Global Average Pooling Layer + Fully Connected layer with Mobile Inverted Bottleneck Convolution | 224×224 |
| **ViT_base_patch16_224** | 12 Transformer layers, Patch size 16×16 | 224×224 |
| **ViT_base_patch32_224** | 12 Transformer layers, Patch size 32×32 | 224×224 |

**^†^**A brief description of each model is provided below.

• ResNet50: ResNet50 addresses the gradient vanishing problem encountered in deep layers by introducing residual learning through skip connections, preserving gradients across layers, and enabling the training of very deep networks.

• Inception-V3: Inception-V3 utilises multi-scale convolutions within parallel modules to effectively learn features at various scales while minimising computational complexity and mitigating overfitting.

• Xception: An extension of the Inception module, Xception separates pointwise convolution and depthwise convolution, allowing for the independent computation of cross-channel correlations and spatial correlations. This design enables the network to learn spatial and channel-specific correlations separately, making it more efficient in processing and learning from image data.

• DenseNet: DenseNet connects the feature maps of each layer to every subsequent layer, facilitating improved feature propagation and significantly enhancing parameter efficiency.

• EfficientNet-B0: EfficientNet systematically balances model scaling across depth, width, and resolution to achieve optimal efficiency. This approach allows the model to deliver superior performance with fewer parameters and computational resources.

• ViT: ViT applies transformers from the natural language processing domain to image classification tasks. By treating image patches as sequence elements, ViT effectively captures both global and local features, demonstrating superior performance compared to CNN architectures while requiring fewer computational resources for training.

Abbreviations: ViT, vision transformer
