# Supplementary material for: AI-augmented differential diagnosis of granulomatous rosacea and lupus miliaris disseminatus faciei: A 23–year retrospective pilot study
Source: PLoS One. 2025 Jun 30;20(6):e0326763. doi: 10.1371/journal.pone.0326763 (PMC12208491; doi:10.1371/journal.pone.0326763)
Supplement: S1 Fig — (DOCX) [file pone.0326763.s002.docx]

**Supporting Information**

**
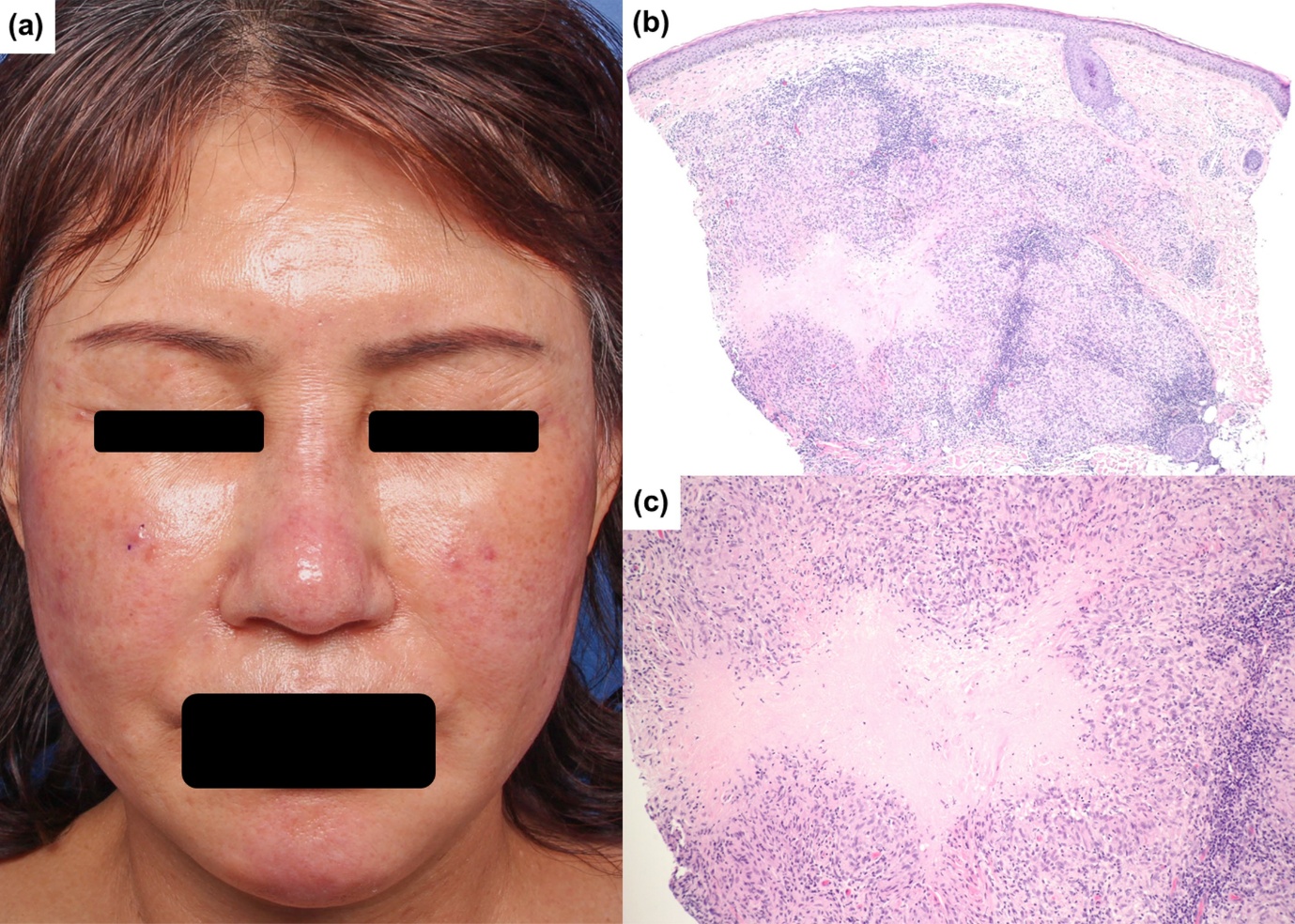
**

**S1 Fig.** A case of lupus miliaris disseminatus faciei associated with nontuberculous *Mycobacterium* infection

(a) A 65-year-old female patient with a history of hypothyroidism visited our hospital with a chief complaint of multiple match-head-sized erythematous papules on both periorbital areas and cheeks, which had persisted for 3 years. She reported undergoing laser treatments, needle aspirations, and using oral medications and topical agents at a local clinic over the past 3 years, without any improvement. Additionally, she mentioned having received unauthorised facial filler injections prior to the onset of the lesions. (b) A biopsy was performed, and the histopathological findings with hematoxylin and eosin staining revealed granulomatous infiltration in the dermis under low-power magnification. (c) Under high-power magnification, caseous necrosis was observed, and based on the clinical and histopathological findings, the patient was diagnosed with lupus miliaris disseminatus faciei. Accordingly, doxycycline was prescribed but resulted in no improvement. The treatment was then switched to isotretinoin, which also failed to show any effect. Consequently, polymerase chain reaction for *Mycobacterium* was performed using a paraffin block obtained from the biopsy. The results indicated "POSITIVE for nontuberculous *Mycobacterium* (NTM)," leading to the initiation of NTM-specific treatment with clarithromycin and levofloxacin. After one month of these medications, all previously existing lesions completely resolved.
