# Supplementary material for: AI-augmented differential diagnosis of granulomatous rosacea and lupus miliaris disseminatus faciei: A 23–year retrospective pilot study
Source: PLoS One. 2025 Jun 30;20(6):e0326763. doi: 10.1371/journal.pone.0326763 (PMC12208491; doi:10.1371/journal.pone.0326763)
Supplement: S2 Fig — (DOCX) [file pone.0326763.s003.docx]

**Supporting Information**


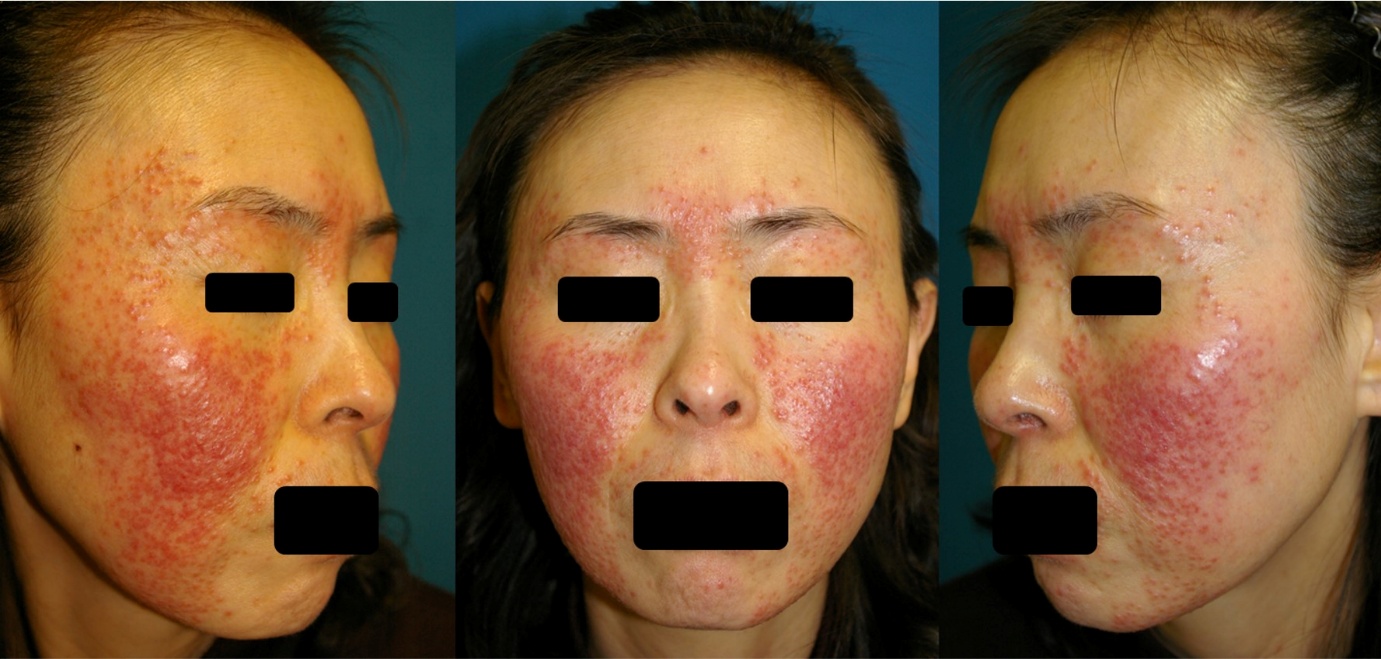


**S2 Fig.** Examples of three photos taken from three different angles of a granulomatous rosacea patient
